# Niao Du Kang Mixture Increases the Expression of mir-129-5p to Relieve Renal Fibrosis

**DOI:** 10.1155/2020/1841890

**Published:** 2020-05-04

**Authors:** Yan-lin Li, Lin-na Liu, Lin Huang, Hai-wen An, Jian-lin Jian, Jie Pang, Fen-na Lin, Wen-qin Yang, Jin-shan Li, Qian Jiang, Hong Wang

**Affiliations:** ^1^Department of Nephrology, Zhongshan Hospital of Traditional Chinese Medicine, Affiliated to Guangzhou University of Chinese Medicine, Zhongshan 528400, China; ^2^Cardiovascular and Cerebrovascular Drugs Research and Development Center, Tianjin Institute of Medical and Pharmaceutical Sciences, Tianjin 300020, China

## Abstract

**Objective:**

To investigate the efficacy of Niao Du Kang (NDK) mixture in renal fibrosis of rats and to explore the mechanism underlying the effect of NDK on renal fibrosis.

**Methods:**

Unilateral ureteral obstruction (UUO) was used to replicate a rat renal interstitial fibrosis model. The drug-administered groups were given 20 ml/kg (NDK-H), 10 ml/kg (NDK-M), and 5 ml/kg (NDK-L) NDK mixture once a day for 21 days beginning 48 hours after surgery. The 24-hour urine protein and serum creatinine (CR) levels in the sham group rats, UUO rats, and NDK mixture-treated rats were measured after the last administration. The pathological changes of rat kidney tissue were observed by HE staining. The degree of fibrosis was observed by Masson's staining and scored. The expression levels of TGF-*β*, *α*-SMA mRNA, and mir-129-5p in kidney were detected by qRT-PCR. HK-2 cells were treated with 5 ng/ml TGF-*β* to induce HK-2 cell fibrosis. The expression levels of TGF-*β*, *α*-SMA mRNA, and mir-129-5p in HK-2 cells were detected by qRT-PCR. TargetScan predicted the target gene of mir-129-5p, HK-2 cells were transfected with mir-129-5p mimic, and an overexpressed mir-129-5p HK-2 cell model was constructed. qRT-PCR was used to detect the expression of PDPK1 mRNA. Western blot was used to detect the expression of PDPK1, AKT, and p-AKT in HK-2 cells induced by TGF-*β* and in UUO rats.

**Results:**

NDK mixture significantly reduced the 24-hour urine protein and CR levels of UUO rats. HE staining showed that the NDK mixture group exhibited a significantly reduced degree of renal interstitial fibrosis. NDK mixture also reduced the expression of TGF-*β* and *α*-SMA, and the middle-dose group showed a better therapeutic effect. In vitro studies showed that NDK mixture-containing serum increased the expression of mir-129-5p to reduce renal fibrosis. In addition, NDK mixture increased the expression of mir-129-5p in vivo. Further studies indicated that mir-129-5p could target PDPKl to reduce its expression. The NDK-containing serum group also exhibited reduced expression of PDPK1.

**Conclusion:**

NDK mixture can significantly improve renal function and improve renal fibrosis in UUO model rats. Furthermore, NDK mixture can inhibit the expression of PDPK1 by upregulating the expression of mir-129-5p and then inhibiting the PI3K/AKT pathway to improve renal fibrosis.

## 1. Introduction

Renal fibrosis is one of the main pathological features of chronic kidney disease and is also the main pathological change and common pathway for renal failure caused by various chronic kidney diseases. In renal fibrosis, overexpression of cytokines leads to proliferation and metabolic disorder of interstitial cells, excess extracellular matrix deposition in the renal interstitium, and destruction and loss of function of renal tissue [1, 2]. Transforming growth factor-*β* (TGF-*β*) is the strongest known fibrogenic factor and plays an important role in the pathogenesis of renal interstitial fibrosis [3]. So far, the mechanism of renal fibrosis has been revealed to be complicated, and some molecular mechanisms still need to be elucidated.

MicroRNAs (miRNAs) are small noncoding RNAs that regulate gene expression at the posttranscriptional level and monitor several biological processes [4]. miRNA research represents a new horizon for the study of the mechanism of renal fibrosis. In-depth research on the roles of miRNAs in renal fibrosis will contribute to the discovery of new drug targets and prognostic biomarkers for this condition [5–7]. mir-129-5p is located in a fragile site on chromosome 7q32, and abnormal expression of this miRNA has been observed in medullary thyroid carcinoma [8], bladder cancer, laryngeal cancer [9], breast cancer [10], gastric cancer [11], osteosarcoma [12], glioma cancer [13], and hepatocellular carcinoma [14]. So far, research on mir-129-5p has mostly focused on tumours, while less research has focused on renal fibrosis. Research on the role of mir-129-5p in renal fibrosis will be very valuable. Our previous research profiled changes in microRNA levels in the HK-2 human kidney proximal tubular cell line with TGF-*β* treatment and identified significantly altered miRNAs, and miR-129-5p is one of the significantly downregulated miRNAs in experimental models. Further research indicated that miR-129-5p suppressed PDPK1 mRNA and protein levels in HK-2 cells. In addition, miR-129-5p inhibited epithelial-to-mesenchymal transition (EMT) via PDPK1 in HK-2 cells [15].

Niao Du Kang (NDK) mixture is a popular medicinal preparation, which consists of rhubarb, salvia, skullcap, safflower, mantle, and other Chinese herbal medicines. It was created to treat the chronic kidney failure, of which the pathogenesis is qi deficiency, blood stagnation, and retention of turbid dampness. NDK mixture removes dampness, dredges the collateral kidney, benefits the qi, and strengthens the spleen [16]. Clinical research has demonstrated that NDK mixture can reduce the serum concentration of creatinine (CR) and improve renal function. Further study indicated that NDK mixture may reduce oxidative stress and inhibit the expression of TGF-*β*1, thus inhibiting peritoneal mesothelial cell injury and peritoneal thickening of the dense layer to protect the peritoneum and delay the process of peritoneal fibrosis [17]. In addition, our previous studies found that NDK mixture can effectively improve renal function and delay the progress of chronic renal failure, but the specific mechanism is not clear. Renal fibrosis is one of the main pathological features of chronic kidney disease. This study was designed to investigate the efficacy of NDK mixture in renal fibrosis and to explore the mechanism underlying the effect of NDK mixture on renal fibrosis.

## 2. Materials and Methods

### 2.1. Cell Line and Animals

The human tubular epithelial cell line HK-2 was purchased from the Chinese Academy of Sciences Shanghai Cell Bank (ATCC number CRL-2190 TM). The cells were cultured in a humidified atmosphere with 5% CO_2_ at 37°C in DMEM/F12 with 10% FBS.

Wistar male rats were purchased from Beijing Vital River Laboratory Animal Technology Co., Ltd. These rats weighed between 200 g and 220 g. The animals were fed in an environment with 20°C–25°C, 40–70% relative humidity, and a 12-hour light-dark cycle.

### 2.2. Models

The rats were allowed to acclimate to the environment for 1 week before the experiment. All experimental procedures were performed in accordance with the International Guidelines for Care and Use of Laboratory Animals. After rats were anaesthetized with 45 mg/kg sodium pentobarbital by intraperitoneal injection, their ureters were ligated and cut between the two ligatures. In the sham operation group, the abdominal cavity of each rat was cut, and the left ureter was freed, but the ureter was not ligated or cut. The positive group was given 30 mg/kg Losartan potassium tablets. The drug-administered groups were given 20 ml/kg (NDK-H), 10 ml/kg (NDK-M), and 5 ml/kg (NDK-L) NDK mixture once a day for 21 days beginning 48 hours after surgery.

### 2.3. Cell Transfection

A mir-129-5p mimic and a negative control (NC) mimic were purchased from RiboBio (Guangzhou, China). HK-2 cells were seeded on 6-well plates (5 × 10^5^ cells/well) and cultured overnight. The cells were transfected with 20 nM mir-129-5p mimic or NC mimic using Lipofectamine 2000 (Life Technologies, USA). The cells were used for Western blot and real-time PCR (qRT-PCR) analysis after transfection for 48 h.

### 2.4. Haematoxylin-Eosin (HE) and Masson's Staining

Rat kidney tissue was fixed in 10% neutral formalin. For HE staining, tissue sections were dewaxed, stained with haematoxylin for 5 minutes, rinsed with running water to remove the remaining stain, differentiated in 0.7% hydrochloric acid ethanol, immersed in 95% ethanol for 30 seconds, stained with alcoholic eosin for 30 seconds, dehydrated, and sealed. For Masson's staining, tissue sections were subjected to conventional dewaxing, stained with haematoxylin for 2 minutes, washed with distilled water, dyed in Lichen red acid magenta for 5 minutes, washed with water, dyed in 1% phosphomolybdic acid for 5 minutes, directly immersed in aniline blue solution for 5 minutes, differentiated in hydrochloric acid ethanol, dehydrated in gradient alcohol, cleared with xylene, and sealed with neutral gum. We scored four aspects: renal tissue fibrosis, inflammatory response, renal atrophy, and tubular dilation. Scores were calculated according to the percentage of the lesion area in the field of vision; 0 points indicated no lesion area, 1 point indicated below 25%, 2 points indicated between 25% and 50%, and 3 points indicated above 50%.

### 2.5. RNA Isolation and qRT-PCR Analysis

Total RNA and miRNA from HK-2 cells and tissue were isolated using a miRNA Purification Kit (CWBio, China). The miRNA was reverse-transcribed into cDNA with a miRNA cDNA Synthesis Kit (CWBio, China). The mRNA was reverse-transcribed into cDNA using a HiFiScript gDNA Removal cDNA Synthesis Kit (CWBio, China). The mRNA and miRNA levels were tested by qRT-PCR using UltraSYBR Mixture (Low ROX) (CWBio, China). The expression of TGF-*β*, *α*-SMA, and 3-phosphoinositide-dependent protein kinase 1 (PDPK1) mRNA was normalized to that of the GAPDH gene, and U6 was used as the internal reference for mir-129-5p. The primer sequences used for qRT-PCR are shown in Table 1. The fold change was calculated using the 2^−∆∆Ct^ method, and the means from three independent experiments were used.

### 2.6. Western Blot Analysis

HK-2 cells and kidney tissue were lysed, and total proteins were extracted with Cell Lysis Buffer for Western and IP (Beyotime, China). The protein concentration was then adjusted to 35 *µ*g/µl with an Enhanced BCA Protein Assay Kit (Beyotime, China). The protein samples were subjected to 10% SDS-PAGE separation and then transferred to PVDF membranes. The PVDF membranes were incubated with antibodies against PDPK1 (1 : 1000, Absin, 0006890201), AKT (1 : 1000, CST, 4691T), p-AKT (1 : 1000, CST, 4060), and *β*-actin (1 : 1000, Boster). Then, the membranes were incubated with peroxidase-conjugated secondary antibodies (1 : 10000, Boster). The protein bands were visualized by enhanced chemiluminescence, and the protein expression was quantitatively analysed by ImageJ.

### 2.7. Statistical Analysis

Analyses were performed using GraphPad Prism 5.0 software. *T*-tests were used to evaluate differences between two groups. One-way analysis of variance (ANOVA) was used to compare means among three or more groups. *P* < 0.05 was considered to indicate a statistically significant result.

## 3. Results

### 3.1. NDK Mixture Ameliorated Renal Fibrosis in Unilateral Ureteral Obstruction (UUO) Rats

**Figure 1:**
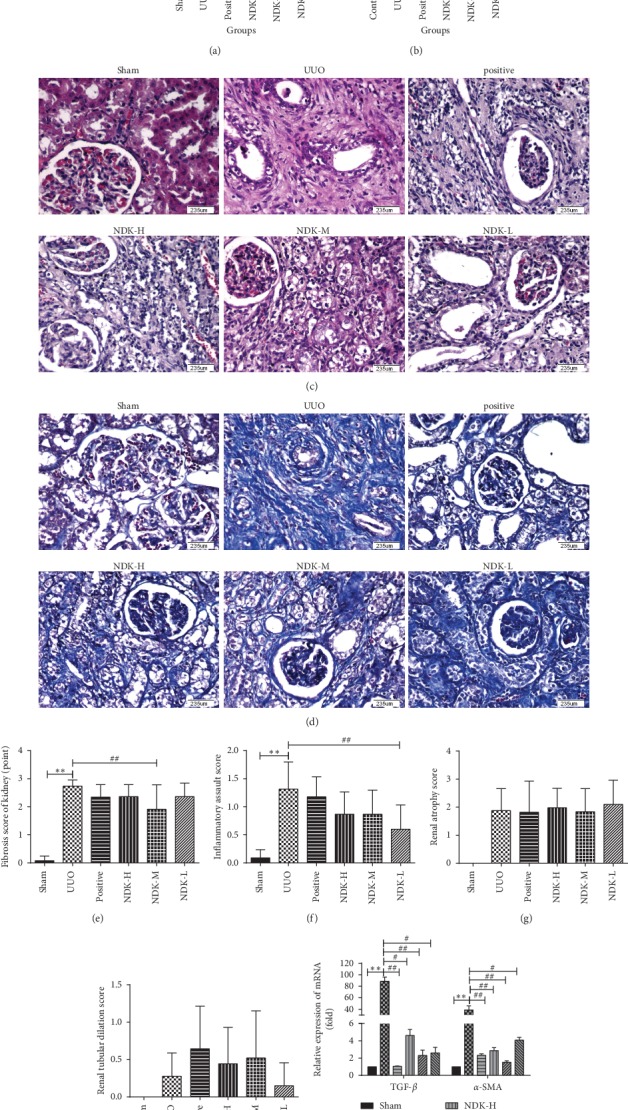
Niao Du Kang mixture improved fibrosis in vitro. (a) 24-hour urine protein content; (b) serum CR content; (c) renal tissue HE staining (200X); (d) renal tissue Masson's staining (200X); (e) renal fibrosis score; (f) inflammatory score; (g) renal atrophy score; (h) tubular dilatation score; (i) the expression of TGF-*β* and *α*-SMA in the sham group, UUO group, positive group, NDK mixture high-dose group, NDK mixture middle-dose group, and NDK mixture low-dose group. The relative expression is homogenized when statistics are performed, and the sham group is defined as 1. ^*∗∗*^*P* < 0.01 vs. the sham group, ^*∗*^*P* < 0.05 vs. the NC group, ^##^*P* < 0.01 vs. the UUO group, and ^#^*P* < 0.05vs. the TGF-*β* group.

We constructed a rat renal fibrosis model by UUO. Then, we determined the 24-hour urine protein levels in UUO rats. The results showed that compared with those of sham group rats, the 24-hour urine protein levels of UUO rats were significantly increased, 36.71 ± 7.948 versus 127.6 ± 22.83. NDK-H (97.40 ± 20.84, *P* < 0.05), NDK-M (59.00 ± 22.31, *P* < 0.05), and NDK-L (59.50 ± 19.80, *P* < 0.01) significantly reduced the 24-hour urine protein levels of UUO model rats (Figure 1(a)). As shown in Figure 1(b), compared with those in the sham group, the serum CR levels in the UUO group were significantly elevated, 20.67 ± 0.6667 *μ*mol/l to 65.83 ± 7.054 *μ*mol/l, and the serum CR levels in the NDK-H (34.89 ± 8.10, *P* < 0.01), NDK-M (32.56 ± 8.71, *P* < 0.01), and NDK-L (30.56 ± 9.23, *P* < 0.01) groups were obviously reduced. According to Figures 1(a) and 1(b), NDK-M had the most significant effect in improving renal function. HE staining (Figure 1(c)) showed that the kidney volume was increased in the UUO group, forming a cystic shape, and the kidney sections showed a substantial pathological phenotype. Under low magnification, the renal pelvis and renal calyces were dilated and deformed, the demarcation of the cortex and medulla was unclear, the renal tubules were extensively atrophied, compensatory expansion was evident, and renal parenchymal cells were reduced significantly in some areas. Under high magnification, renal capsules were widened, glomeruli were disintegrated, renal tubules were atrophied, and epithelial cells were degenerated and necrotic; in addition, the linear structure in the medulla was lost, and this loss was accompanied by infiltration of a large number of inflammatory cells. The pathological manifestations of the NDK-H, NDK-M, and NDK-L groups were alleviated to varying degrees compared with the model group, of which the NDK-M effect was the most obvious. Masson's staining (Figure 1(d)) revealed that the renal interstitium was broadened significantly, that interstitial fibroblasts had proliferated, and that collagen fibres had formed in the UUO group. Semiquantitative analysis showed that the degree of renal interstitial fibrosis in the UUO group was significantly higher than that in the sham group, while the NDK mixture groups exhibited a significantly reduced degree of renal interstitial fibrosis (Figure 1(e)). As shown in Figure 1(f), we scored the inflammatory response of the kidney tissue and found that the UUO model group exhibited an increased inflammatory response, while NDK mixture groups exhibited a reduced inflammatory response. However, NDK mixture groups did not improve kidney atrophy (Figure 1(g)). Although NDK mixture groups improved tubular atrophy, the results were not statistically significant (Figure 1(h)). The NDK-M group had obvious effects in improving renal fibrosis, renal atrophy, and renal tubular dilatation, while inflammation was improved most obvious in the NDK-L group. qRT-PCR showed that the mRNA expression of the key renal fibrosis factors TGF-*β* (88.49 ± 7.54, *P* < 0.01) and *α*-SMA (*P* < 0.01) was significantly higher in the UUO group than in the sham group, while the expression of TGF-*β* (88.49 ± 7.54, *P* < 0.01) and *α*-SMA (38.62 ± 7.41,*P* < 0.01) was decreased in the NDK mixture groups NDK-H (TGF-*β*, 4.62 ± 0.71, *P* < 0.05; DSMA, 2.87 ± 0.34, *P* < 0.01), NDK-M (TGF-*β*, 2.28 ± 0.62, *P* < 0.01; *α*-SMA, 1.51 ± 0.18, *P* < 0.01), and NDK-L (TGF-*β*, 2.61 ± 0.65, *P* < 0.05; *α*-SMA, 4.08 ± 0.32, *P* < 0.05) (Figure 1(i)).

### 3.2. NDK Mixture Increased the Expression of mir-129-5p to Reduce Renal Fibrosis In Vitro

**Figure 2:**
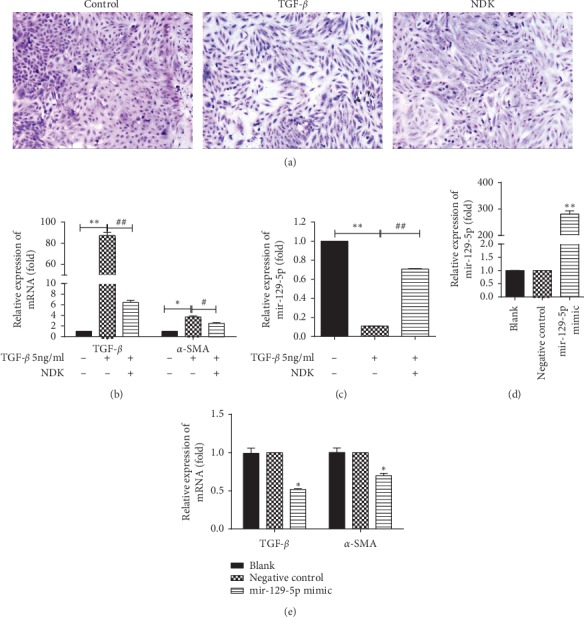
Niao Du Kang mixture increased the expression of mir-129-5p to reduce renal fibrosis in vitro. (a) Giemsa staining of HK-2 cells treated with TGF-*β* and NDK mixture-containing serum (200X); (b) qRT-PCR results of the expression of TGF-*β* and *α*-SMA after treatment with TGF-*β* and NDK mixture-containing serum; (c) qRT-PCR results of the expression of mir-129-5p in HK-2 cells after treatment with TGF-*β* and NDK mixture-containing serum; (d) qRT-PCR results of the expression of mir-129-5p after transfection; and (e) qRT-PCR results of the expression of TGF-*β* and *α*-SMA after transfection. The relative expression is homogenized when statistics are performed, and the sham group is defined as 1. ^*∗∗*^*P* < 0.01, ^*∗∗*^*P* < 0.05, ^##^*P* < 0.05, and ^##^*P* < 0.01.

Next, we further investigated the efficacy of NDK mixture in vitro. HK-2 cells were treated with 5 ng/ml TGF-*β* (PeproTech, 100–21C) for 72 hours to construct a fibrotic model of HK-2 cells. As shown in Figure 2(a), Giemsa staining was used to display cell morphology. qRT-PCR revealed that the mRNA expression of TGF-*β* (87.12 ± 3.26, *P* < 0.01) and *α*-SMA (3.72 ± 0.19, *P* < 0.05) was significantly higher in the TGF-*β* group than in the control group. This indicated that treatment with 5 ng/ml TGF-*β* for 72 hours could successfully induce HK-2 cell fibrosis. Then, HK-2 cells were treated with 8% NDK mixture-containing serum at the same time. Compared with the TGF-*β* group, the TGF-*β* and NDK mixture group exhibited lower mRNA expression of TGF-*β* (6.44 ± 0.39, *P* < 0.01) and *α*-SMA (1.49 ± 0.17,*P* < 0.05) (Figure 2(b)). According to our previous study, it is hypothesized that increases in mir-129-5p may be related to the amelioration of renal fibrosis caused by NDK mixture. Thus, we examined differences in the expression of mir-129-5p among the control, TGF-*β*, and NDK mixture groups by qRT-PCR. The results showed that compared with that in the control group, the expression of mir-129-5p in the TGF-*β*-induced fibrosis group was significantly decreased (0.12 ± 0.08, *P* < 0.01), while the NDK mixture-containing serum increased the expression of mir-129-5p (0.77 ± 0.55, *P* < 0.01) (Figure 2(c)). The mir-129-5p mimic was transfected into HK-2 cells, and qRT-PCR was used to detect the expression of mir-129-5p 48 hours later. The expression of mir-129-5p increased approximately 280-fold after transfection (*P* < 0.01) (Figure 2(d)). This result proved that the overexpression model of mir-129-5p in HK-2 cells was successfully established. qRT-PCR detected the expression of TGF-*β* and *α*-SMA after HK-2 transfection of mir-129-5p mimic. The results are shown in Figure 2(e); mir-129-5p downregulated the expression of TGF-*β* (0.52 ± 0.01, *P* < 0.05) and *α*-SMA (0.70 ± 0.03, *P* < 0.05).

### 3.3. mir-129-5p Targeted PDPK1 to Downregulate Its Expression In Vitro

**Figure 3:**
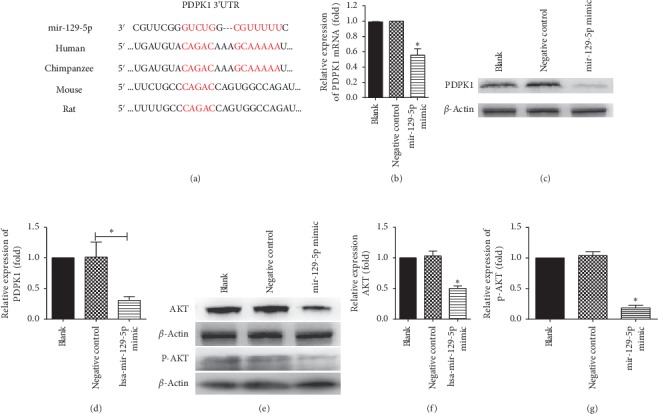
mir-129-5p targeted PDPK1 to downregulate its expression in vitro. (a) TargetScan-predicted sequence alignment of mir-129-5p and PDPK1 3′UTRs from various species; (b) qRT-PCR results of the expression of PDPK1 after transfection; (c) Western blot results of the expression of PDPK1 after transfection; (d) the associated grey value statistics of the expression of PDPK1 after transfection; (e) Western blot results of the expression of AKT and p-AKT after transfection; (f) the associated grey value statistics of the expression of AKT after transfection; and (g) the associated grey value statistics of the expression of p-AKT after transfection. The relative expression is homogenized when statistics are performed, and the sham group is defined as 1. ^*∗∗*^*P* < 0.01, ^*∗*^*P* < 0.05, and ^##^*P* < 0.01.

Next, we further studied the mechanism by which mir-129-5p ameliorates renal fibrosis. First, we found that there was no species specificity between rats and humans by comparing the sequences of mir-129-5p in miRBase. TargetScan Software predicted that PDPK1 was one of the target genes of mir-129-5p, and the binding site is similar among human, chimpanzee, rat, and mouse sequences (Figure 3(a)). We further tested the expression of PDPK1 mRNA and protein in mir-129-5p-overexpressing HK-2 cells, and the results showed that the expression of PDPK1 mRNA and protein decreased greatly after transfection with the mir-129-5p mimic (*P* < 0.05) (Figures 3(b), 3(c), and 3(d)). Since PDPK1 is the key protein in the PI3K/AKT pathway [17], we examined the changes in this pathway, and we found that the pathway was inhibited after overexpression of mir-129-5p (Figures 3(e), 3(f), and 3(g)). Overall, these results suggested that mir-129-5p could target PDPKl to reduce its expression. The NDK mixture-containing serum group also exhibited reduced expression of PDPK1.

### 3.4. NDK Mixture Increased the Expression of mir-129-5p In Vivo

**Figure 4:**
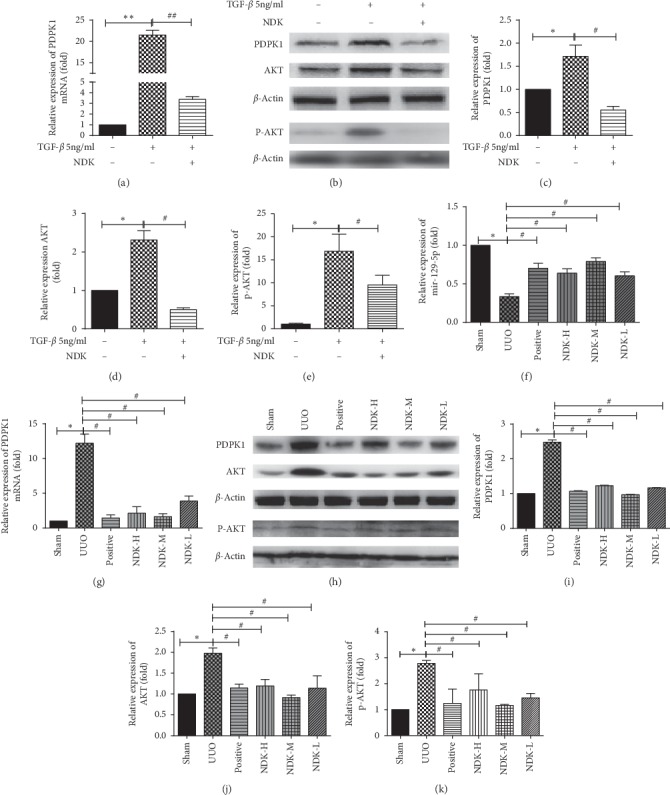
Niao Du Kang mixture increased the expression of mir-129-5p in vivo. (a) qRT-PCR results of the expression of PDPK1 after treatment with TGF-*β* and NDK mixture-containing serum; (b) Western blot results of the expression of PDPK1 and AKT after treatment with TGF-*β* and NDK mixture-containing serum; (c) the associated grey value statistics of the expression of PDPK1 after treatment with TGF-*β* and NDK mixture-containing serum; (d) the associated grey value statistics of the expression of AKT after treatment with TGF-*β* and NDK mixture-containing serum; (e) the associated grey value statistics of the expression of p-AKT after treatment with TGF-*β* and NDK mixture-containing serum; (f) qRT-PCR results of the expression of mir-129-5p in the sham group, UUO group, positive group, NDK mixture high-dose group, NDK mixture middle-dose group, and NDK mixture low-dose group; (g) qRT-PCR results of the expression of PDPK1 mRNA in the sham group, UUO group, positive group, NDK mixture high-dose group, NDK mixture middle-dose group, and NDK mixture low-dose group; (h) Western blot results of the expression of PDPK1 and AKT in the sham group, UUO group, positive group, NDK mixture high-dose group, NDK mixture middle-dose group, and NDK mixture low-dose group; (i) the associated grey value statistics of the expression of PDPK1 in the sham group, UUO group, positive group, NDK mixture high-dose group, NDK mixture middle-dose group, and NDK mixture low-dose group; (j) the associated grey value statistics of the expression of AKT and p-AKT in the sham group, UUO group, positive group, NDK mixture high-dose group, NDK mixture middle-dose group, and NDK mixture low-dose group; and (k) NDK mixture high-dose group, NDK mixture middle-dose group, and NDK mixture low-dose group. The relative expression is homogenized when statistics are performed, and the sham group is defined as 1. ^*∗∗*^*P* < 0.01, ^*∗*^*P* < 0.05, and ^##^*P* < 0.01.

We again verified that NDK mixture can inhibit the expression of PDPK1, AKT, and p-AKT in HK-2 cells (Figures 4(a), 4(b), 4(c), 4(d), and 4(e)). We found that the kidney expression of mir-129-5p was reduced significantly in the UUO group compared with the sham group, while the expression of mir-129-5p was increased in the NDK-H, NDK-M, and NDK-L groups, especially the NDK-L group (Figure 4(f)). qRT-PCR also reflected the increase in the expression of PDPK1 mRNA in the UUO group, which was attenuated in the NDK-H, NDK-M, and NDK-L groups (Figure 4(g)). The Western blot results indicated that the expression of PDPK1 was significantly increased in the UUO group and decreased in the NDK-H, NDK-M, and NDK-L groups (Figures 4(h) and 4(i)), similar to the findings for the other PI3K/AKT pathway members (Figures 4(h), 4(j), and 4(k)). We found that PDPK1 inhibition was most pronounced in the NDK-M group, and the expression of AKT and p-AKT was most downregulated. In conclusion, we further demonstrated in vivo that NDK mixture could inhibit the expression of PDPK1 by upregulating the expression of mir-129-5p and then inhibiting the PI3K/AKT pathway to improve renal fibrosis. NDK-M had the most obvious effect.

## 4. Discussion

Renal fibrosis is a hallmark of end-stage renal disease and is a major cause of therapeutic failure. Previous research has shown that TGF-*β* is the key mediator of renal fibrosis. It is associated with inflammation, cell reproduction, apoptosis, and mutation. Studies have shown that EMT is the key process involved in renal interstitial fibrosis. The main manifestation is the loss of expression of cell adhesion molecules, decreased epithelial markers, and increased stromal markers. Subsequently, epithelial cells have the morphological characteristics of mesenchymal cells [18]. TGF-*β* can stimulate the expression of collagen, fibronectin, and proteoglycan and promote the production of extracellular matrix (ECM). Furthermore, TGF-*β* can increase the activity of tissue inhibitor of metalloproteinases (TIMP) and decrease the activity of matrix metalloproteinase-2 (MMP-2) to inhibit ECM degradation, which plays a very important role in the development of EMT [19]. TGF-*β* is involved in renal fibrosis through multiple pathways, such as the TGF-*β*/Smad signalling pathway, p38 mitogen-activated protein kinase (MAPK) signalling pathway, and extracellular signal regulated kinase (ERK) signalling pathway. Additionally, *α*-SMA is a marker protein of renal interstitial fibrosis. Decreasing the expression of SMA can inhibit the activation of fibroblasts, thus reducing the excessive accumulation of ECM and effectively delaying renal fibrosis [20]. In this study, we constructed rat renal fibrosis model by UUO and detected the key renal fibrosis factors TGF-*β* and *α*-SMA. We found that the mRNA expression of TGF-*β* and *α*-SMA was significantly higher in the UUO group than in the sham group. Additionally, we constructed a TGF-*β*-induced renal tubular epithelial fibrosis model with HK-2 cells. qRT-PCR revealed that the mRNA expression of TGF-*β* and *α*-SMA was significantly higher in the TGF-*β* group than in the control group.

The pathological foundations for the development of renal fibrosis and the syndrome of blood stasis in Chinese medicine are closely related. NDK mixture has the effect of removing dampness, dredging kidney collateral, benefiting qi, and strengthening the spleen. Studies have shown that NDK mixture can reduce the level of serum CR in patients with chronic renal failure and improve renal function, thereby delaying the development of end-stage renal disease. In addition, studies have shown that NDK mixture can play an antifibrosis role and improve the function of nephropathy in multiple ways. NDK mixture can reduce the levels of 24-hour urinary protein, serum CR, blood urea nitrogen, and microalbumin and increase the levels of haemoglobin and serum calcium in rats with chronic renal failure [16]. Our previous study showed that NDK mixture can reduce acute tubular necrosis induced by glycerol. A possible underlying mechanism is that NDK mixture can reduce oxygen free radical injury, protect antioxidant and ATP enzymes, and relieve calcium overload [21]. Further research shows that NDK mixture may have the effect of alleviating oxidative stress and inhibiting the expression of TGF-*β*1, thereby inhibiting the injury of peritoneal mesothelial cells and the thickening of the peritoneal dense layer, thus protecting the peritoneum and delaying peritoneal fibrosis [17]. Salvia miltiorrhiza, rhubarb, safflower, and astragalus are the main components of NDK mixture. Salvia miltiorrhiza can protect the renal function in rats of aristolochic acid nephropathy (ANN) by obviously ameliorating the anemia, reducing the level of serum CR and lessening the excretion of 24-hour urine protein and urine NAG enzyme [22]. Furthermore, salvia miltiorrhiza may reduce the expression of ACE, promote the synthesis of ACE2, inhibit the activity of AngII and downregulate the overexpression of TGF-*β*1 and PAI-1, so as to alleviate renal interstitial fibrosis in ANN rats [23]. Emodin, the main effective component of rhubarb, can reduce the spindle change induced by IL-1*β*, downregulate the expression of *α*-SMA, upregulate the expression of cytokeratin, and inhibit the transdifferentiation of renal tubular epithelial cells induced by IL [24]. Rhein, another important component of rhubarb, can reduce the levels of TGF-*β*1 and the renal cortex homogenate lipid marker MDA while increasing the levels of SOD and antioxidant enzyme CAT, thus improving renal interstitial fibrosis caused by UUO [25]. In addition, safflower can improve renal interstitial fibrosis. The difference is that safflower reduces the degree of renal interstitial fibrosis in UUO rats mainly by reducing the expression of fibronectin (FN) and collagen I (ColI) [26]. Astragalus has been found to ameliorate renal fibrosis via the TGF-*β*/Smad and NF-*κ*B signalling pathways in vivo and in vitro [27]. In this study, we found that NDK mixture could obviously reduce the levels of 24-hour urine protein and serum CR in UUO model rats. This is consistent with previous studies. Moreover, NDK mixture can significantly alleviate renal interstitial fibrosis and decrease the levels of TGF-*β* and *α*-SMA. In general, the middle-dose group showed a better therapeutic effect. NDK mixture-containing serum could also reduce the expression of TGF-*β* and *α*-SMA in fibrotic cells in vitro.

miRNA-129 is mainly encoded by two genes, miRNA-129-1 and miRNA-129-2. Research on miRNA has mainly focused on tumours. miRNA-129 produces three mature bodies, miRNA-129-5p, miRNA-129-1-3p, and miRNA-129-2-3p. miRNA-129-5p is considered to carry out the function of miRNA-129. Thus far, research on miRNA-129 has mainly focused on cancer. Cui's study indicated that miRNA-129-3p downregulation promotes EMT, in vitro invasion, and in vivo metastasis of hepatocellular cancer (HCC) cells via activation of PI3K/Akt and p38-MAPK signalling pathways, partially by targeting Aurora-A [28]. Brest's research showed that miR-129-5p accentuated the antiproliferative effects of other cancer drugs such as etoposide or human *α*-lactalbumin, resulting in lethality for tumour cells and highlighting a miRNA-driven cell death mechanism [29]. However, only a few studies have examined the relationship between miR-129 and renal fibrosis. Xiao's study demonstrated that mir-129-5p is related to renal fibrosis and acts as an inhibitor of TGF-*β*1 during renal fibrosis, while mir-129-5p directly targets Smad interacting protein 1 (SIP1) and the 3′UTR of the gene SOX4, inhibits its posttranscriptional activity, and decreases the expression of renal fibrosis-related proteins E-cadherin and Claudin-1 [30]. Furthermore, our previous research proved that miR-129-5p significantly declined in the HK-2 human kidney proximal tubular cell line with TGF-*β* treatment. PDPK1 is a potential target gene of miR-129-5p, and luciferase assay analysis identified PDPK1 as a new direct target gene of miR-129-5p. Further research indicated that miR-129-5p suppressed PDPK1 mRNA and protein levels in HK-2 cells. In summary, miR-129-5p functions to inhibit renal fibrosis and EMT in HK-2 cells via PDPK1 [15].

In this study, we found that the pharmacodynamics of NDK mixture were related to the upregulation of mir-129-5p expression. After overexpressing mir-129-5p in HK-2 cells, we found that mir-129-5p relieved HK-2 cell fibrosis. This result is consistent with the observed inhibition of EMT in breast cancer cells after overexpression of mir-129-5p [31]. Further research showed that mir-129-5p targeted PDPK1 to downregulate its expression. PDPK1 is a regulator of the PI3K/AKT pathway [32]. We examined the changes in this pathway, and we found that the pathway was inhibited after overexpression of mir-129-5p in vitro, which had the same effect as the NDK mixture-containing serum. An in vivo study also demonstrated that NDK mixture can inhibit the expression of PDPK1 by upregulating the expression of mir-129-5p and then inhibiting the PI3K/AKT pathway to improve renal fibrosis. Therefore, further studies are necessary to explore the mechanism of action of mir-129-5p.

## 5. Conclusions

NDK mixture can significantly improve renal function and improve renal fibrosis in UUO model rats. Furthermore, NDK mixture can inhibit the expression of PDPK1 by upregulating the expression of mir-129-5p and then inhibiting the PI3K/AKT pathway to improve renal fibrosis.

## Figures and Tables

**Table 1:** Primer sequences used for the qRT-PCR analysis.

Application	Oligonucleotides	Sequences (5′-3′)
TGF-*β*	Forward	AGCAACAATTCCTGGCGATACCTC
	Reverse	TCAACCACTGCCGCACAACTC
*α*-SMA	Forward	TGCTGGACTCTGGAGATGGTGTG
	Reverse	CGGCAGTAGTCACGAAGGAATAGC
PDPK1	Forward	CTTCGTCCTCCTCCTCACACTCC
	Reverse	AGCCTGCTTCTCCAACAACAACC
GAPDH	Forward	ACGGCAAGTTCAACGGCACAG
	Reverse	CGACATACTCAGCACCAGCATCAC
mir-129-5p	Forward	CGCTTTTTGCGGTCTGG
	Reverse	AGTGCAGGGTCCGAGGTATT
U6	Forward	CTCGCTTCGGCAGCACA
	Reverse	AACGCTTCACGAATTTGCGT

## Data Availability

The data used to support the findings of this study are included within the article.
